# Cost effectiveness of malaria vector control activities in Sudan

**DOI:** 10.1186/s12936-024-04900-7

**Published:** 2024-03-15

**Authors:** Sami M. HasapAla, Rasha S. Azrag, Osama M. Awad

**Affiliations:** 1 Department of Environmental Health, Faculty of Public Health, Shendi University, Shendi, Sudan; 2 Vector Genetics and Control Laboratory, Department of Zoology, Faculty of Science, University of Khartoum, Khartoum, Sudan; 3 Department of Environmental Health, High Institute of Public Health, Alexandria University, Alexandria, Egypt

**Keywords:** Insecticide residual spraying, Long-lasting insecticidal nets, Larval source management, Operational cost, Entomological surveillance

## Abstract

**Background:**

Malaria vector control activities in Sudan rely largely on Long-Lasting Insecticidal Nets (LLINs), Indoor Residual Spray (IRS) and Larval Source Management (LSM). The present study attempted to determine cost effectiveness of inputs and operations of vector control interventions applied in different environmental settings in central and eastern Sudan, as well as their impact.

**Methods:**

The inputs utilized and cost of each vector control activity, operational achievements and impact of the applied malaria vector control activities; IRS, LLINs and LSM were determined for eight sites in Al Gazira state (central Sudan) and Al Gadarif state (eastern Sudan). Operational costs were obtained from data of the National Malaria Control Program in 2017. Impact was measured using entomological indicators for *Anopheles* mosquitoes.

**Results:**

The total cost per person per year was $1.6, $0.85, and $0.32 for IRS, LLINs and LSM, respectively. Coverage of vector control operations was 97%, 95.2% and 25–50% in IRS, LLINs and LSM, respectively. Vectorial capacity of malaria vectors showed statistically significant variations (P < 0.034) and ranged 0.294–0.65 in areas implemented LSM in comparison to 0.097–0.248 in areas applied IRS and LLINs, respectively. Both indoor and outdoor biting *Anopheles* mosquitoes showed noticeable increase that reached 3–12 folds in areas implemented LSM in comparison to areas implemented IRS and LLINs. Annual malaria prevalence was 13.1–21.1% in areas implemented LSM in comparison to 3.20%, 4.77% in areas implemented IRS and LLINs, respectively.

**Conclusion:**

IRS and LLINs are cost effective control measures due to adequate inputs and organized process. However, the unit cost of LSM intervention per outcome and subsequently the impact is hugely affected by the low coverage. The very weak support for implementation of LSM which includes inputs resulted in weakness of its process and consequently its impact. Implementation of LSM by local government in urban settings is challenged by many factors the most important are maintenance of adequate stable level of funding, un-adequate number of well trained health workers, unstable political and administrative conditions and weak infrastructure. These challenges are critical for proper implementation of LSM and control of malaria in urban settings in Sudan.

**Supplementary Information:**

The online version contains supplementary material available at 10.1186/s12936-024-04900-7.

## Background

Sudan is one of the countries with a high malaria burden in sub-Saharan Africa, with an estimated annual malaria incidence of 27.4 cases per 1000 population and a case fatality rate of about 2 deaths per 100,000 [1]. It is the third largest country by area in Africa and includes a wide range of ecological strata. Therefore, matrix of malaria eco-epidemiology included desert fringe, low stable endemic control, hypo and mesoendemic classes, urban class, irrigated schemes and major dams in addition to emergency and complex situations [2]. Malaria transmission shows a seasonal and unstable pattern and depends on rainfall except in urban settings and irrigated schemes where the transmission is around the year.

Sudan adopted multiple prevention vector control strategies which included indoor residual spraying (IRS) in targeted areas with irrigation schemes, long-lasting insecticidal nets (LLINs), larval source management (LSM) using temephos in urban settings. In addition, environmental management (EM) is carried in urban settings and include different activities, such as intermittent irrigation, repair of broken water pipes and open of rain drain canals [3, 4].

Considerable investments have been put into the scaling-up of malaria control interventions following Global Fund to Fight AIDS, Tuberculosis and Malaria resources since 2002 and the global movement for Scale Up For Impact (SUFI). This was reflected in many efforts which included the support of the LSM in urban settings by the government [5].

Monitoring and evaluation are essential components of malaria vector control activities and enables assessment of the efficacy of its vector control operations and adjust its policies to make the most efficient use of scarce resources [6, 7]. It has two inter-related components: (1) monitoring of programmatic implementation (process), and (2) evaluation of interventions (outcome and impact). Impact measures the reduction observed in transmission of the disease through defined indicators whose calculation is based on epidemiological and entomological surveillance [8, 9].

This study attempted to cost the input and operations of vector control interventions adopted in urban and rural settings in central and eastern Sudan as well as its impact.

## Methods

### Study area

The present study was carried out in eight sites located et al. Gazira state at central Sudan and Al Gadarif state at eastern Sudan.

Al Gazira State lies in the rich Savanna region between latitude 13°–15° and the longitude 32.5°–34°. It is administratively divided into seven localities. The climate of Wad Madani locality, which includes the capital city of Al Gazira state, is considered as hot and dry most of the year. Maximum temperature exceeds 40 °C on average during summer season (April to May) and drops to below 15 °C during winter season (December to February). Most of the rainfall is received during July to October and the mean annual rainfall may exceeds 350 mm.

Al Gadarif State is located between longitudes 33°–36° East, and latitudes 12º–15º North. It shares an international border with Ethiopia to its east. It is admistratively divided into five localities. It is characterized by semi-tropical and dry savannah climate, with a maximum temperature of 41° C in April and May and the mean annual rainfall is about 400 mm per year (July–October).

### Selection of study sites

**Fig. 1:**
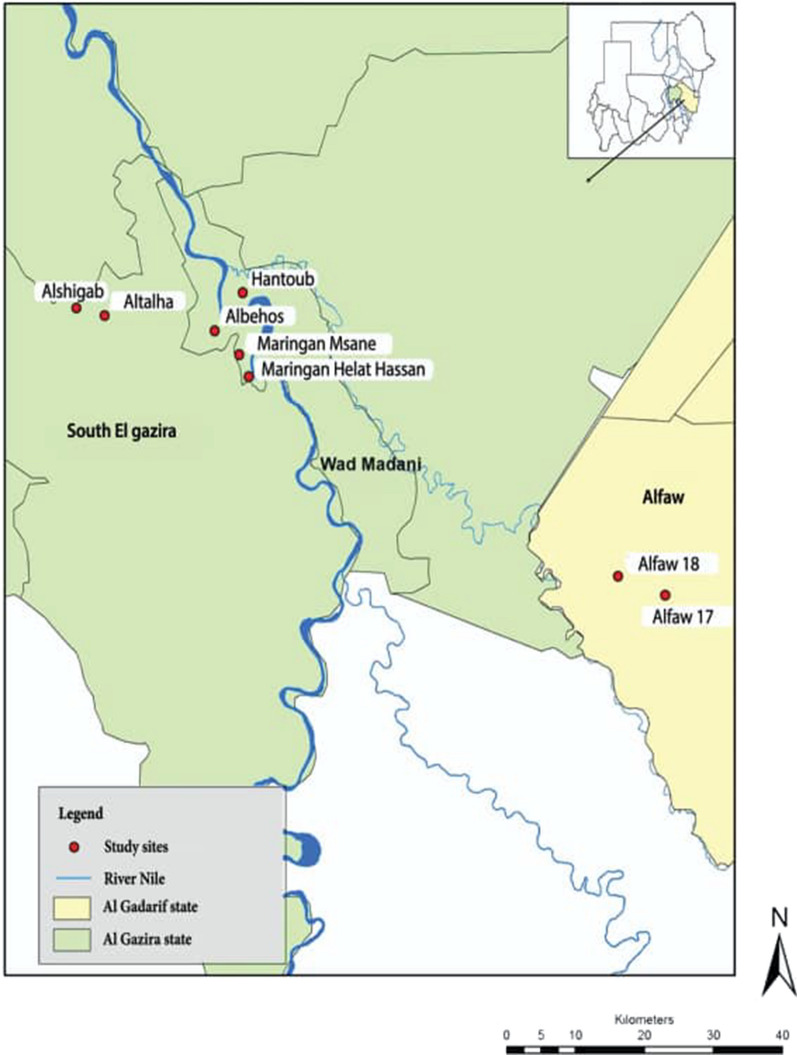
Geographical locations of study sites

According to the malaria control strategy in 2017 LSM is conducted in urban areas and LLINs and IRS were conducted in rural areas. Four urban sites (Maringan Helat Hassan, Hantoub, Maringan Msane and Albehos district allocated in Wad Madani city/Al Gazira state) were chosen to represent LSM activity. According to their proximity to River Maringan Helat Hassan and Hantoub districts (both applied LSM only) were combined together and Maringan Msane and Albehos districts (both applied LSM plus EM) were also combined together to represent control activities in urban settings supplemented with EM and referred to as LSM and LSM + EM, respectively. Another two rural areas (Alshigab and Altalha villages allocated in South Al Gazira locality) were combined together to represent IRS activity. These two rural sites are part of Al Gazira agricultural Scheme which is the largest agricultural Scheme in Sudan and the majority of the inhabitants work in agriculture and animal husbandry. In addition, two rural areas (Alfaw17 and Alfaw18 villages) which are located in Alfaw locality et al. Gadarif state were combined together to represent LLINs activity. The most important economic activities of the population of these two sites are agriculture and animal husbandry. Figure 1 shows geographical locations of study sites.

### Estimation of operational costs

Input utilized and their cost and outcomes in terms of operational achievements (*i.e*. coverage and quality, timing, frequency, pesticides used, dosage, quantity, equipment, performance and days need) were obtained from monitoring and evaluation data of National Malaria Control Programme (NMCP) and Malaria Vector Control Unit (MVCU) in the local government after their permission. The collected financial data covered the campaigning of IRS, LLINs and LSM activities during 2017.

Ingredient approach was used, and inputs of intervention were identified, valued and classified for each vector control activity. The cost of IRS was divided into two categories, capital costs and recurrent costs according to Creese & Parker [10], and Yukich et al. [11], then the share of each village/district was calculated from total cost of locality. The costs of LLINs and LSM activities was adopted the same way used for estimation of operational costs of IRS All costs converted to US-$ according to exchange rate in 2017.

In addition, costs of one of the most common EM activities (environmental modification through drainage of rain drain canals by the beginning of the rainy season) in urban areas was estimated. This was estimated in Maringan Msane and Albehos districts (El Gazira State). The costs of IRS, LLINs, LSM and EM are provided in Additional files 1, 2 and 3.

### Output of vector control interventions

#### Entomological indicators

Because of the limitation of the MCP data in terms of estimation of all entomological indicators of malaria transmission and the presence of numerous missing data we conducted a cross sectional entomological surveys for *Anopheles* mosquitoes (adults and immatures) during the rainy season (August to October, 2017) for three days/ month. Entomological surveys were carried out in each of the selected village/district using Pyrethroid Spray Sheet Collection (PSC) method in 20 houses [8, 12], human baited CDC Light Trap collection (LTC) method (indoor and outdoor) in two houses and Exit Window Trap (EWT) method in two houses [12]. Larval collection was performed in 20 stations (10 fixed and 10 spot-check stations) in every village/district according to [12]. Data was collected using designated forms.

#### Morphological and molecular identification

Adults and larvae were identified using morphological keys [13]. 50 random samples of adult females were identified using species specific identification using PCR technique [14].

#### Malaria prevalence

Malaria prevalence in 2017 was calculated from the documents of health centres in each village/district.

#### Data analysis

The entomological indicators of malaria transmission included indoor resting density (IRD), man-biting rate (MBR), human blood index (HBI), sporozoite rate (SP), entomological inoculation rate (EIR), parity rate (PR), longevity (Long), sporogony cycle (N) and vectorial capacity (VC) and they were calculated as described in the literature [12, 15–21].

IRD = Number of mosquitoes collected using PSC/Number of houses surveyed using PSC, HBR = F/W (M: Is the man- biting rate, F: Is the total number of freshly fed (FF) mosquitoes of the particular species, W: Is the total number of human occupants in houses used for collection.)

 Resting habit = KHD/NPM (k: a correction value of 1.16, H: human blood index, D: indoor resting density (total number of females *Anopheles gambiae* collected divided by number of houses used from the spray-sheet collection) and N: average number of persons per house (household size)),

 HBI = Number of female mosquitoes positive for human blodd/Total number of mosquitoes fed analysed. In this study the sources of mosquito blood meals was estimated as 0.6 for *An. gambiae* depending on the previous studies conducted in Sudan and Ethiopia [15, 16].

SR = Number of mosquitoes with* Plasmodium sporozoites*/Total number of mosquitoes examined × 100,

 EIR =   Sporozoite rate ×   man-bitinng (%)/100,

 proportion parous = FF + HG+ G/UF + FF + HG + G,* P*= 3√ proportion parous, longevity = 1/−lnp, = T/(t–t min)  (n: duration of sporogony cycle; T: 111, 105, 114 for r*Plasmodium falciparum*, *Plasmodium vivax* and *Plasmodium malariae*, respectively; t: actual average temperature in degrees centigrade and t min = 16 for *P. falciparum* and *P. malariae* and 14.5 for *P. vivax*),

VC  = ma2*pn*/ − *lnp* (m: density of vector in relation to man (bites/person/night); a: number of blood meals taken on man per vector per day (human blood index multiplied by 0.5, if a gonotrophic cycle of three days is assumed); p: daily survival rate and n: incubation period) Metrological data needed for estimation of certain entomological indicators was obtained from the Sudanese Metrological Authorities (SMA).

The data of costs, inputs utilized and operational achievements and entomological indicators of malaria vectors (mean values of entomological parameters) was calculated using Windows Excel and SPSS programs (Version 16). One way analysis of variance was applied to estimate p values of mean entomological indicators across different vector control activities.

## Results

### Inputs utilized and operational achievements

Table 1 shows inputs utilized and operational achievements of IRS, LLINs, LSM1 and LSM2 activities. IRS Spray operations were conducted according to the guidelines adapted from the WHO protocols [22]. The LLINs, LSM and EM operations were conducted according to the [23, 24] respectively. Coverage of EM was estimated based on the fact environmental modification includes drainage, filling, land levelling and transformation and impoundment margins. However, only one activity was carried out by responsible authorities (cleaning of rain drain canals) in addition to the fact that most rain drain canals are not tightly covered to prevent breeding of mosquitoes.

**Table 1:** Inputs utilized and operational achievements for malaria vector control programs in 2017

**Operational monitoring**	**Achievements and inputs utilized**
**IRS**	
Coverage	97% of the target rooms in two villages
Timing	By the beginning of the rainy season (August, 2017) and at the end of the rainy season (December, 2017)
Frequency	Two rounds per year
Pesticides	(ficam) bendiocarb
Dosage	200mg\m^2^
Quantity	180.2 kg per year
Equipment	Hudson pump (good condition) + uniform + tools of pump spear parts
Performance	-The spraying was conducted daily -End of the campaign report was done -Pre-training of workers, technicians and supervisors was performed as well as data entering personnel
Days needed	8 days a year (2 villages, 2 rounds for 2days each village)
**LLINs**	
Coverage	95.2% of the targeted persons
Timing	Early before the rainy season, 2017
Frequency	once every three years
Pesticides	Deltamethrin
Dosage	50 mg/m^2^, Rectangular – 190 * 180 * 150 cm Mesh – 24/cm^2^, 20 washes (approx. 3 years)
Quantity (number of nets distributed)	9,846 nets
Equipment	No
Performance	Distribution of nets on all targeted persons
Days needed	2 days every three years (one day for each village)
**LSM**	
Coverage	25–50% of the targeted sites (water bodies)
Timing	All year long
Frequency	Once per week
Pesticides	Temephos 50% ECW/V
Dosage	10 cc Temephos in 10 litters water
Quantity	140 L per year
Equipment	Hudson pump (good condition) + uniform + tools of pump spear parts
Performance	- Wad Madani City was divided into sectors - Health workers performed spraying operations (coverage the mosquito breeding sites by insecticide) - End of the work report was done and delivered to office of malaria control
Days needed	52 days a year (one day a week for a year) for each district
**EM**	
Coverage	Approximately 30% of the target sites at each district
Timing	Before and during rainy season
Frequency	once a year
Pesticides	NO
Dosage	NO
Quantity	NO
Equipment	Loader, boklen, tractor, monitoring car, drill equipment and suction pump
Performance	-Simple environmental modification and manipulation approaches were conducted annually (mainly opening of rain drain canals) - End of the season report was done
Days needed	15 days a year (for two districts)

### Financial costs

**Fig. 2:**
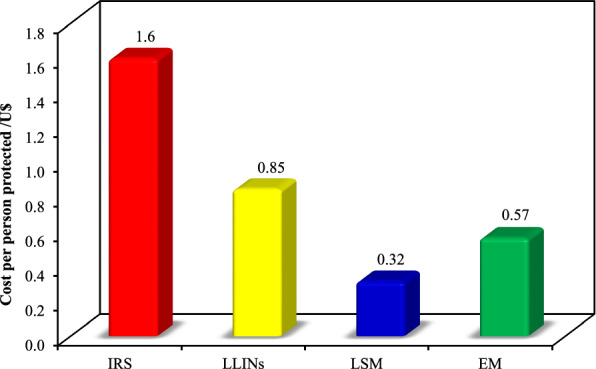
Cost per person in US$ of different malaria vector control activities

The highest cost per person per year was 1.6 US$ for IRS activity followed by 0.85 US$ for LLINs while the lowest cost per person per year was 0.32 US$ for LSM activity (Fig. 2). Environmental management activity conducted before the rainy season included only cleaning of rain drains canals and cost per person per year was 0.57 US$.

### Entomological indicators of malaria transmission

A total of 9,679 adult mosquitoes were collected over three months during the rainy season (August to October 2017) by three methods of collection (PSC, LTC, EWT) in eight study sites. 66.3% of the total collection was *An. gambiae* complex, 1.6% *Anopheles rufipes* and 0.1% *Anopheles pharoensis*. 50 random females of *An. gambiae* complex from all study sites were identified using species specific PCR techniques and results showed that 66% of *An. gambiae* complex were *Anopheles arabiensis*.

Table 2 shows entomological indicators of *An. arabiensis* in areas implemented different vector control interventions. Relatively highest values of IRD and MBR were in areas implemented LSM. Statistically significant variations (P < 0.034) were in VC with the lowest value (0.09) in areas implemented LLINs activity while high values (0.650) was in areas implemented LSM activity. For immature, the higher densities of *Anopheles* larvae were found in areas implemented LSM activity (8.9 ± 1.4–10.9 ± 6.9) compared to other areas implemented IRS or LLINs (7.4 ± 0.69, 5.7 ± 2.01, respectively).

**Table 2:** Entomological indicators of malaria transmission during August – October 2017 in areas implement different vector control activities in eastern and central Sudan

Entomological parameters	IRS	LLINs	LSM	LSM + EM	P
	Mean ± STD				
IRD	9.816 ± 3.25	5.441 ± 0.907	4.775 ± 1.768	19.308 ± 15.91	0.022*
MBR	0.332 ± 0.243	0.118 ± 0.079	0.247 ± 0.101	0.548 ± 0.584	0.165
HBI(estimated)	0.6	0.6	0.6	0.6	1.000
RH	0.915 ± 0.092	0.874 ± 0.073	0.819 ± 0.032	0.809 ± 0.072	0.061
SR	0.138	0.138	0.138	0.138	1.000
EIR	0.0458 ± 0.033	0.0163 ± 0.010	0.0341 ± 0.0139	0.0757 ± 0.080	0.165
PR	0.766 ± 0.077	0.800 ± 0.064	0.850 ± 0.0338	0.865 ± 0.081	0.069
P	0.914 ± 0.030	0.927 ± 0.025	0.947 ± 0.0125	0.952 ± 0.029	0.064
Long	12.7 ± 5.448	14.4 ± 4.948	19.3 ± 5.095	26 ± 18.140	0.133
N	8.43 ± 0.314	9.1 ± 0.389	8.43 ± 0.314	8.43 ± 0.314	0.005*
VC	0.248 ± 0.304	0.097 ± 0.080	0.294 ± 0.1298	0.650 ± 0.510	0.034*
Larval density/dip	7.4 ± 0.69	5.7 ± 2.01	10.9 ± 6.80	8.9. ± 1.40	0.400

IRD Indoor resting density, MBR Man-biting rate, HBI Human blood index, RH Resting habit, SP Sporozoite rate, EIR Entomological inoculation rate, PR Parity rate, P Daily survival rate, Long Longevity, N Sporogony cycle, VC Vectorial capacity

^*^: Statistically significant at p ≤ 0.05

### Biting pattern of malaria vectors

Table 3 shows the biting pattern of *Anopheles* spp during the rainy season (August to October, 2017) from 6 pm to 6 am in areas implemented different control activities. The peak of both indoor and outdoor *Anopheles* bites in all areas was between 2 and 4 am.

**Table 3:** Biting pattern of *Anopheles* mosquitoes collected from areas implemented different vector control activities

Time		IRS T.N		LLINs T.N		LSM T.N		LSM + EM T.N	
		Indoor	Outdoor	Indoor	Outdoor	Indoor	Outdoor	Indoor	Outdoor
Night	6 – 8 pm	11	11	3	0	0	2	37	21
	8 – 10 pm	16	7	7	4	4	14	17	61
	10 – 12 am	12	13	25	6	10	18	37	183
Morning	12 – 2 am	9	23	12	21	52	59	49	219
	2 – 4 am	20	12	23	19	124	64	141	230
	4 –6am	6	5	9	11	32	78	49	176
Total (%)		74 (3.7)	71(3.6)	79(4.0)	61(3.1)	222(11.3)	235(11.9)	330(16.8)	890(45.3)

T.N. Total Number

While lower numbers of indoor and outdoor biting *Anopheles* mosquitoes were recorded in areas implemented IRS (3.7% and 3.6% respectively) and LLINs (4.0% and 3.1% respectively). Both indoor and outdoor biting *Anopheles* mosquitoes showed noticeable increase that reached 3–12 folds in areas implemented LSM in comparison to areas implemented IRS and LLINs.

### Prevalence of malaria disease

Areas implemented IRS or LLINs showed the lowest prevalence of malaria (4.77% and 3.20%, respectively) however, malaria prevalence was 3–7 folds higher in areas implemented LSM (13.12% and 21.1%, respectively) (Table 4).

**Table 4:** Prevalence of malaria disease in four sites applied IRS, LLINs, LSM and combination of LSM and EM as malaria vector control strategies in 2017

Month	IRS		LLINs		LSM		LSM + EM	
	No	%	No	%	No	%	No	%
January	30	0.39	69	0.33	237	1.26	91	2.2
February	28	0.36	64	0.31	161	0.86	54	1.3
March	17	0.22	43	0.21	191	1.02	53	1.27
April	16	0.21	33	0.16	191	1.02	44	1.06
May	23	0.30	34	0.16	166	0.88	42	1.01
June	20	0.26	36	0.17	115	0.61	49	1.18
July	24	0.31	56	0.27	214	1.14	60	1.44
August	43	0.56	66	0.32	257	1.37	92	2.20
September	43	0.56	81	0.39	215	1.14	105	2.52
October	47	0.61	71	0.34	236	1.25	119	2.85
November	38	0.50	52	0.25	218	1.16	93	2.23
December	37	0.48	42	0.20	267	1.42	75	1.80
Total	366	4.77	660	3.20	2468	13.12	877	21.1
Total number of Population	7.674		20.607		18.813		4.164	

## Discussion

Vector control is critical for the reduction and interruption of malaria transmission as it breaks the transmission cycle, thereby reducing morbidity and mortality. Both IRS and LLINs can dramatically reduce the burden of malaria by killing adult female mosquitoes when they come to take human blood meals [25]. Although the primary goal of such interventions is the reduction of human/vector contact rather than reduction of the vector population, these control methods may also suppress the local vector population under certain circumstances [26]. In contrast, larval control measures are intended to reduce malaria transmission indirectly by reducing the vector population density near human habitations [27].

In 2017, Sudan adopted multiple prevention vector control strategies which included) IRS in targeted areas with irrigation schemes with seven targeted states, LLINs in twelve states, LSM using temephos on weekly basis and function in 110 urban settings in 18 states [2]. This study attempted to determine cost effectiveness of on-going different malaria vector control activities taking place in Sudan by NMCP in 2017. Both IRS and LLINs control interventions are completely funded by the Global Fund, WHO and other international agencies, and LSM is funded by the local government. The operational achievements of both IRS and LLINs activities showed 95.2% -97% coverage. Cost of LLINs reported in this study (0.85 US$) was greater than cost per person protected/year (0.32 US$) reported in Ghana [28] and less than the cost per individual protected per year (0.87 US$) reported in Sri Lanka [29]. IRS activity costs per person per year was ($ 1.6 US$) and it is less than the value of 3.86 US$ reported from southern Mozambique [30] and sub-Saharan Africa [11] and greater than the cost reported from Kenya valued at 0.86 US$ [31]. Both LLINs and IRS activities were the most cost-effective compared to LSM according to epidemiological (prevalence of malaria) and entomological indicators.

LSM activity had low cost (cost per person per year of 0.32 US$ and accordingly the lowest outcome of operational achievements in comparison to IRS and LLINs activities. However, the unit costs of intervention per outcome in low coverage hugely vary for the cost at high coverage (which was the case for both IRS and LLINs). The calculation of unit cost of LSM intervention is only valid for its context including the reported low level of coverage and higher cost of LSM in high coverage (which may reach double the unit price) is expected. In terms of financial costs this result is less than the cost per person protected per year in Tanzania—Dar el Salaam (0.94 US$) and west Kenya high lands ($1.50) [32] and approximately equivalent to the value (0.49 US$) reported in Sri Lanka [29]. The reported low cost of LSM may be due to the reported low coverage 25–50% which was accompanied with high density of immature malaria vectors (9.8–10.9 larvae/dip) during the rainy season. It is well known that LSM is intrinsically reliant upon careful, continuous performance management of large implementation teams based on feedback from monitoring systems for adult mosquito densities [33].

Low coverage of LSM highlights the challenges in applying it in Madani locality and other similar urban settings in Sudan. Madani locality as well as most urban settings in Sudan suffers from unplanned urbanization. These settings has numerous uncovered rain drain canals, water storage practices of inhabitants and aggregation of other indoor/outdoor water bodies suitable for breeding of malaria vectors might complicate application of LSM. Assessed the impact of urbanization on malaria transmission in sub-Saharan Africa and presented evidence that urbanization allows for increased rates of disease transmission, large numbers of larval development sites due to construction of new settlements, water storage practices and limited methods for disposal of wastewater and refuse. All previously listed factors are well known in Madani locality as well as other urban settings in Sudan [34, 35].

Challenges for proper application of LSM include: (1) insufficient financial support from state governments that adequately covers all operational activities, (2) severe lack of well-trained health workers, (3) unstable political conditions that accordingly results in unstable administrative process, (4) very weak infrastructure, (5) weak inter and intra-sectoral collaboration between health authorities and other related sectors, (6) absence of involvement of local communities, (7) long use of single larvicide with some reports from urban settings documenting the appearance of resistance to temephos larvicide in urban cities [34]. These listed factors are critical for the control of malaria in urban settings in Sudan.

EM activities are carried out before the beginning of the rainy season in urban settings in Sudan. All these settings are characterized by unplanned urbanization. The estimated cost of the only EM activity carried out (cleaning of rain drain canals) was $0.57 per person per year. However, the reported EM cost exceeds the value of ($0.24) reported in Sri Lanka [28]. In addition, it is clear that this estimation is significantly affected by the the fact that other EM activities were not considered by responsible authorities. It is critical that responsible authorities of EM activities consider all possible activities instead of reliance upon one activity (cleaning of rain drain canals) which do not executed perfectly to prevent breeding of mosquitoes.

The vectorial capacity (VC) of mosquito species is an index used to compare actual or potential vectorial importance of particular species with that of another or to evaluate vector control activities within a given area. This study showed that the vectorial capacity of *An. arabiensis* for the all vector control activities in the four study areas exceeded 0.01, which is considered as the minimum value required for the vector to maintain malaria transmission [36]. The mean value of VC (0.24) for IRS activity, reported in this study is similar to the VC value (0.24) reported from central Sudan [37].

Efficiency and effectiveness of malaria vector control activities specifically IRS and LLINs depending on the biting pattern of malaria vectors [38]. Many studies highlighted the shift in biting behavior of Anopheline mosquitoes during or after the implementation of malaria vector control activities and may further influenced by the availability of potential hosts in different locations [39]. The present study showed that the peak indoor biting of malaria vectors in areas implemented IRS at 2 am to 4 am and the peak of outdoor biting at 12 am to 2 am. This result is in agreement with the biting peak of malaria vector (*An. gambiae*) outdoor at (12 am to 2 am) reported in Tanzania [40]. For areas implemented LLINs** t**he peak indoor biting of malaria vectors was at 10 pm to 12 am. This result parallel with findings of a study conducted In Equatorial Guinea for the biting peaks of *An. gambiae* [41]. The shown peak biting rate of malaria vectors out door at (12 am to 2 am), is similar to the biting peak for *An. gambiae *sensu stricto from 12 am to 2 am (both indoors and outdoors) from Tanzania [39]. The study finding showed that number of malaria vectors caught outdoors (222 and 890) was more than indoor (235 and 330) in areas implemented LSM during the rainy season.

The reported number of malaria cases of total population from routine surveillance was used as core indicators for tracking of malaria vector control activities in all study areas. Malaria prevalence peaked in all study areas during the rainy season. This study presented a mean of malaria prevalence of 13.2 − 21.1% in areas implemented LSM activity. It is less than the mean malaria incidence (40%) and (56.7%) in a multi-country study undertaken in Sudan, Kenya, India, Cameroon and Benin and Ethiopia [42, 43]. Depending on health facilities reports to assess the impact of community interventions is associated with many challenges that may not be accurate to reflect the true situation of the disease burden in that community. However, all health centres use microscopic examination as the diagnostic tool for malaria disease and there are no other health centre facilities in the study sites. The present data showed higher prevalence of malaria cases reported from areas implemented LSM control intervention. Data obtained from health centres highlight the continuous transmission of malaria during the hot dry season (for all interventions) and the urgent need for proper malaria vector control methods during these seasons, specifically for LSM.

## Conclusion

IRS and LLINs are cost-effective methods of malaria control. Application of LSM in urban settings in Sudan is challenged by many factors, including financial, political, weak infrastructure and human capacity and administrative instability. The very weak financial and logistic support for implementation of LSM resulted in weakness of process and significantly affects its impact. It is very critical that local government overcome such challenges for the control of malaria disease in urban settings.

### Supplementary Information

**Additional file 1. **Financial costs included in the analysis of the LLIBN and IRS control activities.

**Additional file 2. **Financial costs included in the analysis of the LSM and EM activities.

**Additional file 3: Table S4.5.** Expenditure for IRS malaria vector control activity in Alshigab and Altalha Villages in two rounds per year (2017, US$). **Table S4.6.** Expenditure for LLIBN malaria vector control activity in Alfaw 17 and Alfaw 18 Villages , in one round per 3 years, (2017 US$). **Table S4.7.** Expenditure for LSM malaria vector control activity in Maringan Helat Hassan and Hantoub Districts per year, (2017 US$). **Table S4.8.** Expenditure for EM malaria vector control activity in Maringan Msane and Albehoth Districts per year, (2017 US $).

## Data Availability

The data used or/and analyzed during this study are available from the corresponding author on reasonable request.
