# Supplementary material for: Cost effectiveness of malaria vector control activities in Sudan
Source: Malar J. 2024 Mar 15;23:80. doi: 10.1186/s12936-024-04900-7 (PMC10943848; doi:10.1186/s12936-024-04900-7)
Supplement: Supplementary file 1 — Additional file 1. Financial costs included in the analysis of the LLIBN and IRS control activities. [file 12936_2024_4900_MOESM1_ESM.docx]

Additional 1: Financial costs included in the analysis of the LLIBN and IRS control activities.

|  | **LLINs** | **IRS** |
| --- | --- | --- |
| **Capital Cost** | Buildings | Buildings |
|  | Vehicles | Vehicles |
|  | Computer + printer | Sprayers |
|  |  | Computer + printer |
|  |  |  |
| **Recurrent Cost** | Fixed –personnel | Fixed –personnel |
|  | Temporary – personnel | Temporary – personnel |
|  | Supervision | Supervision |
|  | Uniform | Uniform |
|  | Tools | Tools |
|  | Transportation | Transportation |
|  | Spear parts + fuel | Spear parts + fuel |
|  | Training | Training |
|  | Impregnated nets | Insecticides |
|  | Printing+ data entering | Printing+ data entering |
