# Supplementary material for: Cost effectiveness of malaria vector control activities in Sudan
Source: Malar J. 2024 Mar 15;23:80. doi: 10.1186/s12936-024-04900-7 (PMC10943848; doi:10.1186/s12936-024-04900-7)
Supplement: Supplementary file 2 — Additional file 2. Financial costs included in the analysis of the LSM and EM activities. [file 12936_2024_4900_MOESM2_ESM.docx]

Additional ‎2: Financial costs included in the analysis of the LSM and EM activities.

|  | **LSM** | **EM** |
| --- | --- | --- |
| **Capital Cost** | Buildings | Buildings |
|  | Vehicles | Boklin + tractor |
|  | sprayers | Computer + printer |
|  | Computer+ printer |  |
| **Recurrent Cost** | Personnel | Fixed– personnel |
|  | Supervision | Temporary – personnel |
|  | Uniform | Supervision |
|  | Tools | Uniform |
|  | Transportation | Fuel |
|  | Fuel | Transportation |
|  | Training | Training |
|  | Insecticides | Insecticides |
|  | Printing + data entering | Printing+ data entering |
|  |  |  |
