# Supplementary material for: Cost effectiveness of malaria vector control activities in Sudan
Source: Malar J. 2024 Mar 15;23:80. doi: 10.1186/s12936-024-04900-7 (PMC10943848; doi:10.1186/s12936-024-04900-7)
Supplement: Supplementary file 3 — Additional file 3: Table S4.5. Expenditure for IRS malaria vector control activity in Alshigab and Altalha Villages in two rounds per year (2017, US$). Table S4.6. Expenditure for LLIBN malaria vector control activity in Alfaw 17 and Alfaw 18 Villages , in one round per 3 years, (2017 US$). Table S4.7. Expenditure for LSM malaria vector control activity in Maringan Helat Hassan and Hantoub Districts per year, (2017 US$). Table S4.8. Expenditure for EM malaria vector control activity in Maringan Msane and Albehoth Districts per year, (2017 US $). [file 12936_2024_4900_MOESM3_ESM.docx]

**Expenditures of vector control activities**

**Table 4.5: Expenditure for IRS malaria vector control activity in Alshigab and Altalha Villages in two rounds per year (2017, US$).**

| **Costs** | **Alshigab Village** | **Altalha Village** | **Alshigab and Altalha villages** |
| --- | --- | --- | --- |
|  | **Cost /year US$** | **Cost /year US$** | **Cost /year US$** |
| **Capital cost** | | | |
| Vehicle | 38.7 | 37.1 | 75.8 |
| Building | 38.7 | 37.1 | 75.8 |
| Sprayers | 8.9 | 8.6 | 17.5 |
| Computer + printer | 1.3 | 1.3 | 2.6 |
| **Sub total** | **87.6** | **84.1** | **171.7** |
| **Recurrent cost:** | | | |
| Fixed –personnel | 1,221 | 1,221 | 2,442 |
| Temporary – personnel | 2,710 | 2,710 | 5,420 |
| Supervision | 330 | 330 | 660 |
| Uniform | 261.7 | 165.7 | 427.4 |
| Tools | 8.4 | 5.5 | 13.9 |
| Transportation | 711 | 711 | 1422 |
| Spear parts + fuel | 16 | 10.7 | 26.7 |
| Training | 422.7 | 422.7 | 845.4 |
| Insecticides | 695 | 357.4 | 1,052.4 |
| Printing+ data entering | 7.7 | 5.1 | 12.8 |
| **Total / year** | **6,471 US$** | **6,023US$** | **12,494 US$** |

**Table 4.6: Expenditure for LLIBN malaria vector control activity in Alfaw 17 and Alfaw 18 Villages , in one round per 3 years, (2017 US$).**

| **Costs** | **Alfaw Village 17** | **Alfaw Village 18** | **Alfaw 17 & Alfaw 18 Villages** |
| --- | --- | --- | --- |
|  | **Cost /year US$** | **Cost /year US$** | **Cost /year US$** |
| **Capital cost** | | | |
| Vehicle | 95.6 | 251.3 | 346.9 |
| Building | 95.6 | 251.3 | 346.9 |
| Computer + printer | 3.2 | 8.4 | 11.6 |
| **Sub total** | **194.4** | **511** | **705.4** |
| **Recurrent cost:** | | | |
| Fixed – personnel | 706.2 | 1,855 | 2,561.2 |
| Temporary – personnel | 216.8 | 569.7 | 786.5 |
| Supervision | 60.2 | 158.3 | 218.5 |
| Uniform | No | No | No |
| Transportation | 107.3 | 276 | 383.3 |
| Spear parts + fuel | 23.8 | 62.6 | 86.4 |
| Training | 14.5 | 38 | 52.5 |
| Impregnated nets | 8,498 | 22,714 | 31,212 |
| Printing+ data entering | 3.2 | 25.2 | 28.4 |
| **Total per three years** | **9,824 US$** | **26,210 US$** | **36,034 US$** |
| **Total / year** | **3,274.7 US$** | **8,736.7 US$** | **12,011.4 US$** |

**Table 4.7: Expenditure for LC malaria vector control activity in Maringan Helat Hassan and Hantoub Districts per year, (2017 US$).**

| **Costs** | **Maringan Helat Hassan** | **Hantoub** | **Maringan helat Hassan and Hantoub Districts** |
| --- | --- | --- | --- |
|  | **Cost /year US$** | **Cost /year US$** | **Cost /year US$** |
| **Capital cost** | | | |
| Vehicle | 69.4 | 81.33 | 150.73 |
| Building | 69.4 | 81.33 | 150.73 |
| Sprayers (85) | 5.3 | 7.88 | 13.2 |
| Computer + printer | 2.3 | 3.5 | 5.8 |
| **Sub total** | **146.4** | **174** | **320.4** |
| **Recurrent cost:** | | | |
| Personnel | 1,939.3 | 2,909 | 4,848.3 |
| Supervision | 16.7 | 25 | 41.7 |
| Uniform | 49.3 | 73.9 | 123.2 |
| Tools | 5 | 7.6 | 12.6 |
| Transportation | 138.9 | 208.3 | 347.2 |
| Fuel | 27 | 40.6 | 67.6 |
| Training | 68.9 | 145.8 | 214.7 |
| Insecticides | 148 | 222.3 | 370.3 |
| Printing+ data entering | 7 | 10.4 | 17.4 |
| **Total / year** | **2,546.5 US$** | **3,817 US$** | **6,363 US$** |

**Table 4.8: Expenditure for EM malaria vector control activity in Maringan Msane and Albehoth Districts per year, (2017 US $).**

| **Costs** | **Maringan Msane** | **Albehoth** | **Maringan Msane and Albehoth Districts** |
| --- | --- | --- | --- |
|  | **Cost /year US $** | **Cost /year US $** | **Cost /year US $** |
| **Capital cost** | | | |
| Boklin +tractor | 123.45 | 246.9 | 370.35 |
| Building | 2.7 | 5.4 | 8.1 |
| Computer + printer | 2.3 | 4.6 | 6.9 |
| **Sub total** | **128.5** | **256.9** | **385.4** |
| **Recurrent cost:** | | | |
| Fixed– personnel | 512.5 | 1,025 | 1,537.5 |
| Temporary – personnel | 0 | 0 | 0 |
| Supervision | 30.1 | 56.8 | 86.9 |
| Uniform | 0 | 0 | 0 |
| Fuel | 55.6 | 102 | 157.6 |
| Transportation | 23.2 | 46.4 | 69.6 |
| Training | 38.9 | 77.8 | 116.7 |
| Insecticides | 0 | 0 | 0 |
| Printing+ data entering | 6.9 | 13.8 | 20.7 |
| **Total / year** | **795.7 US$** | **1,578.9 US$** | **2,374.6 US$** |
